# American Dental Association

**Published:** 1863-08

**Authors:** 


### AMERICAN DENTAL ASSOCIATION.

This body met in Philadelphia the 28th of July, and continued its session four days, and was largely attended. There were present over fifty delegates. Nearly all the delegates appointed by local societies were in attendance, which we consider a good indication. There was much work done, and it was well done ; every member seemed impressed with the idea that he had something to do, and he was determined to do it. The reports and essays, for the most part, evinced labor, research and study, and the discussions upon them were highly interesting and instructive.

And further upon this subject, we can not do better than quote from the pen of Prof. McQuillen:

"It is with feelings of more than ordinary pleasure that attention is directed to the fact that, contrary to what might have been anticipated at such times, viz., the rebel invasion of the North, active operation of the conscription act, and the riotous conduct of Northern sympathizers with the Rebellion, the third annual meeting of this Association, which was held in Philadelphia during the latter part of July, was, due to the exertions and self-sacrificing devotion of earnest laborers in different parts of the country, largely attended by representations from the various local dental societies.

"The attendance at the previous meetings was very limited, and the prospects were that such would continue to be the case for some time to come, for the Association has had many difficulties to contend with. It was organized at a period when the causes which led to the present national troubles were culminating, and these have shook every institution in the country to their base, and crushed many a noble enterprise which has not had sufficient vitality to stand such an ordeal.

"In addition to these causes, the objects and aims of the organization have been misunderstood by some, and studiously and persistently misrepresented by others, and in this way many desirable persons have been induced to hold aloof from it.

"For a brief period, such efforts may meet with a certain amount of success in opposition to any enterprise, but sooner or later, if the movement is a good one, and its promoters and supporters have the requisite ability, energy, consistency, and determination to maintain its vitality during the early and most trying period of existence, a reaction correspondingly beneficial to the organization will eventually take place in the minds of those who have perhaps been most opposed to it, when they become fully and clearly acquainted with its objects, aims, and capabilities.

"The individual or institution which escapes, or when tried, can not bear misrepresentation and opposition, has very little of the temper or backbone which is demanded in effecting the progressive movements of the age. Such things may crush the weak, the timid, and the irresolute, but they only serve to invigorate and strengthen those who are firm of purpose, and whose principles of action are based upon the unwavering convictions that their cause is not only just and proper, but its promulgation and establishment are imperatively demanded by the requirements of the times.

"The proper response, therefore, to misrepresentation is *work;* earnest, effective, and unmistakably progressive in its character. 'By its fruit shall a tree be known.' And so it is with men and institutions. To move steadily forward in a laudable undertaking, animated by a sincere desire to increase the boundaries of human knowledge and the avenues for the relief of suffering humanity, indifferent alike to unmerited praise or censure, neither weakened by the one nor intimidated by the other, is far better than to waste time, talents, and energies in useless and unprofitable controversies. The air may be thrown into vibrations by a few mouthfuls of articulate sound by those who place a higher estimate on their own opinions than others are willing to accord to them; or page after page of nonsense may be published by some poor fellow who is constitutionally and incurably afflicted with *cacœthes scribendi*, but what then? sooner or later the vibrations of the air will cease, and the published matter will be used in various ways as waste paper. The time and occasion may arrive when it becomes proper and necessary to make a simple statement of *facts* in refutation of erroneous assertions, and then it should be done in a clear, unmistakable, and incontrovertable manner.

"The American Dental Association has passed through such an ordeal, and come out of it with an indication of vitality and strength which gives a fair promise of future usefulness and prosperity.

"Resting as this organization does upon a representative basis, its advantages and beneficial influences are not confined merely to its members, but extend to every local society connected with it, and through them to the community at large. The members of the Association not only come together annually, each bringing with him the past year's experience in practice, and the discussion of subjects in the local societies, but also on returning to their respective sections, they carry with them to their fellow-members of the local societies the rich results of their conference with the delegates to the national association. The national and local societies in this way react upon each other, and with decided advantage to the community, the profession, and associated effort generally.

"In addition to this, no organization can exercise a more powerful influence upon dental education and dental literature, or bring about promptly and effectually such modifications and improvements as may be demanded in each."

